# Development and Implementation of a Multidimensional Narrative Support System for Emergency Nurses: An Action Research

**DOI:** 10.1155/2024/6162718

**Published:** 2024-07-27

**Authors:** Hairong Yu, Yue Deng, Li Gui

**Affiliations:** ^1^ School of Nursing Naval Medical University (Second Military Medical University), Shanghai 200433, China; ^2^ Basic Medical Department Naval Medical University (Second Military Medical University), Shanghai 200433, China

## Abstract

**Background:**

Emergency nurses may experience various fatiguing duties and suffer more pressure, so it is necessary to promote their professional or work-related quality of life, especially during tough periods.

**Aim:**

To describe the development of the multidimensional narrative support system and explore its effectiveness and feasibility among emergency nurses.

**Design:**

An action research was conducted in Shanghai, China, from 2022 to 2023.

**Methods:**

Two cycles of action research were adopted in the emergency departments of two tertiary general hospitals in Shanghai, China. A total of 20 and 13 emergency nurses from different posts participated in each cycle. Multiple methods and tools, such as validated instruments, self-designed questionnaires, and individual and focus group interviews, were used to collect short- and long-term data. The EQUATOR guidelines on reporting action research were used as the guideline for this study.

**Results:**

The multidimensional narrative support system was gradually modified to promote its feasibility. Involved emergency nurses actively participated in different activities and proposed relatively positive and satisfying remarks. Quantitative data showed the significantly instant or long-lasting improvements in emergency nurses' professional and work-related quality of life, as well as their self-compassion, and perceived social support. Various categories were summarized about the participants' experiences, perceived long-term effects, and suggestions on the multidimensional narrative support system.

**Conclusion:**

The multidimensional narrative support system integrates narrative methods and psychological activities and provides multidimensional support for emergency nurses. In spite of various challenges, the system shows good feasibility and significant influences on emergency nurses' well-being and quality of life. *Implications for Nursing Management.* The multidimensional narrative support system may action as a novel intervention for the overall improvements of clinical emergency nurses, which can be recommended to other populations with targeted modifications and training, so as to achieve good generalization in different departments.

## 1. Introduction

The emergency department is a stressful working environment where nurses may experience physically and psychologically fatiguing duties and suffer more time pressure [1]. Even well clinically trained, emergency nurses also admitted difficulties in interpersonal relationships, high level of responsibility, and absence of feedback for work [2]. In recent years of COVID-19, emergency nurses were directly involved in the pandemic, which made them susceptible to infection and higher levels of stress [3]. Therefore, emergency nurses may be at risk of negative experiences and emotions and report low levels of professional or work-related quality of life [3, 4].

Professional quality of life is a general construct and involves positive and negative aspects of work life, including compassion satisfaction, compassion fatigue, and burnout [5]. Professional quality of life may not only influence nurses' job performance but also affect their physical and mental health [6, 7]. Work-related quality of life refers to the quality of experience and condition of life as people interact in the employee-employer relationship [8], which was revealed to correlate with individual's physical and emotional wellbeing, the quality of services, and outcomes related to patients [9]. Consequently, it is necessary for nursing researchers and managers to promote emergency nurses' professional or work-related quality of life, especially in the context of tough periods.

In recent years, various interventions and programs have been developed for nurses to achieve their overall well-being. The Compassion Fatigue Resiliency Program is a structured, comprehensive training program to prevent compassion fatigue and improve resilience, which has been implemented among different populations [10, 11]. Pehlivan and Güner [10] designed a randomized controlled trial with a large sample of oncology-haematology nurses and found that compassion satisfaction scores of nurses participating in this program were significantly higher than those who did not. The mindfulness-based stress reduction (MBSR) incorporates meditation, body and breath awareness, and mind-body practices, and various studies revealed that MBSR was effective to manage stress, enhance resilience and compassion satisfaction, and reduce burnout [12, 13]. Considering the time commitment of traditional MBSR, a 3-minute mindfulness intervention was explored with 3 times of breathing exercise per day over 4 weeks, demonstrating significant reductions in compassion fatigue [14], which proved the efficacy of small MBSR on reducing the physiological stress of nurses [12]. Inspired by the above-mentioned activities, Fu et al. [15] developed an educational program integrating compassion fatigue resiliency, mindfulness respiration, and relatives and friends' support, which had short- and medium-term positive effects on nurses' professional quality of life, as well as physical and mental health. Considering the significance of compassion in nursing profession, McVicar et al. [16] developed a compassionate mind model-based curriculum and found that the 12-month course for health visiting students might reduce their fears of compassion, as well as compassion fatigue.

In addition to the psychological interventions, some physical relaxations and herb therapies were also used for promoting nurses' professional quality of life. The progressive muscle relaxation exercises were intervened for nurse managers, and these exercises over 8 weeks were revealed to significantly decrease their levels of compassion fatigue and burnout [17]. Considering the effects of patchouli oil inhalation on reducing healthy adults' stress and sympathetic nerve activity, Shin et al. [18] designed a randomized controlled trial to investigate the effects of patchouli inhalation, which reported reduced levels of stress and increased compassion satisfaction among emergency nurses.

During the COVID-19 pandemic, a randomized controlled experimental study was carried out to investigate the effects of motivational messages sent to emergency nurses for 21 days, and higher job satisfaction and communication skills and lower compassion fatigue were reported [19]. A 12-week intervention of structured debriefing sessions was explored among trauma health care professionals experiencing patient death, and three questions were asked at every debriefing around the relationship-based care model [20]. Besides using storytelling or narrative alone to address psychosocial stress in health care professionals, expressive arts interventions are gaining their popularity and were found to have more favorable outcomes [21]. Phillips et al. [22] combined storytelling, reflective writing, music, and psychoeducation to aid oncology nurses on awareness and expression of work-related emotions and indicated that the storytelling through music was feasible and acceptable to address work-related emotions and psychosocial stress.

This study focused on those individual, work-related, and perceived supportive factors of emergency nurses' professional quality of life, which were reported in the hierarchical multiple regression and path analysis models [7, 23] and took advantage of narrative or storytelling to develop the multidimensional narrative support system (MNSS) from the perspectives of individual, expert, and colleague, aiming to improve emergency nurses' professional and work-related wellbeing. To make the MNSS theoretically solid, several relevant models or theories were referenced as the theoretical bases of this study.

Reflective writing is a process of personal and creative writing for reflection on patient care and socialization into medicine [24]. Shapiro et al. [24] put forward its conceptual model, in which the first phase is individual and solitary writing, requiring writers' personal voice, imagination, creativity and reflection, as well as the loss of certainty and authority. During the writing, writers should also recognize and respond to those emotions triggered in patient care [24]. The second phase in the model is small-group reading and discussion, which could lead to the acknowledgement of vulnerability and risk-taking for the writers, and the exercise in witness and mindfulness for the listeners [24]. The two processes may contribute to practitioners' professional development and well-being, together with improved patient care [24]. In view of above-mentioned applicable elements and goals, the reflective writing model was selected to design the primary steps of the MNSS.

Expressive writing is an individual-focused intervention, which aims to promote emotional expression during adaptation to stressful situations, and consequently improves overall health from the emotional, social, biological, and cognitive levels [25]. In the standard protocol of expressive writing, participants can focus on their deepest thoughts and feelings about a negative life experience that they choose and write for 15 to 20 min for several sessions over a few days [26]. Although the writings were usually brief exercises over a limited period, the process definitely invited people to think about their emotions and lives in general. Relatively loose and vague requirements on expressive writing times and intervals present good suitability to guide the writing phase of the MNSS. These features may provide emergency nurses with relaxing atmosphere to express their emotions freely and help to heal their ailments [25, 26].

This study aimed to adopt the action research design to develop and refine the MNSS and further explore its effectiveness and feasibility. Research questions of this study were as follows: how was the process of MNSS developed and refined by two cycles of action research? What effects did the MNSS have on emergency nurses? What were the experiences of emergency nurses who participated in the MNSS?

## 2. Materials and Methods

### 2.1. Design

The mixed-method action research was used to develop and implement the MNSS, as well as explore its effects among emergency nurses. Action research can be used to describe and explain social situations while implementing a change intervention aimed at improvement [27, 28]. Cyclical activities including planning, acting, observing, and reflecting were conducted for two rounds exactly from July, 2022, to February, 2023, in this study. The observation and reflection phases helped to develop new initiatives and address the knowledge-action gap [29]. Enhancing the QUAlity and Transparency Of health Research (EQUATOR) guidelines on best practice in reporting action research was used as the guideline for this study [30]. Ethical approval was obtained from the institutional review board of the university.

### 2.2. Settings and Participants

This study was performed in the emergency departments of two tertiary general hospitals in Shanghai City, China. The emergency departments consist of multiple sections and provide comprehensive emergency patient care. Nurses in the emergency departments undertake the posts in the resuscitation room, infusion room, intensive care unit, observation room, and precheck and triage table, etc. Two sampling strategies of purposive and convenient sampling were used for each research cycle. The eligible criterion was the nurses working in the emergency departments for at least one year. After the lecture on *“Emotion Management and Narrative Writing”* and the introduction of MNSS for nurses in the emergency department, eligible nurses were invited by nursing managers for participation. In the first cycle, the recommendations of nurse managers were referenced to select participants and then the participants completed the preintervention survey. In the second cycle, the sequence of recruitment and preintervention survey was changed, and those nurses reporting the score of compassion fatigue ≥2.5 in the survey were invited by the nursing manager as potential participants.

### 2.3. Research Team

The research team consisted of nursing researchers and assistants, nurse managers, and psychologists. The researchers and assistants were responsible for the whole process of developing and applying the NMSS, as well as assigning the tasks to other team members. Nurse managers mainly assisted the recruitment of participants and the time schedule of activities. The psychologists participated in and organized the group training.

### 2.4. Development and Application of MNSS

#### 2.4.1. Planning and Development

Considering the relevant factors of professional quality of life among emergency nurses, such as those negative individual experiences, the levels of working stress, self-compassion, and perceived social support [7, 23], the above-mentioned conceptual model of reflective writing and the method of expressive writing were utilized to develop the MNSS. Consequently, the draft of MNSS was developed with the aims to develop emergency nurses' self-compassion, improve their professional and work-related quality of life, and gain organizational support. The multidimensional activities in the draft included five procedures over a period of 4 weeks, which were lecture, narrative writing (individual dimension), group discussion (colleague dimension), interview analysis (expert dimension), and group training. An expert meeting was organized to revise the draft, and 8 nursing experts with the background of nursing humanities, nursing management, and emergency and critical care nursing were invited to discuss the rationality and feasibility of the draft. The 90-minute recording of the expert meeting was transcribed literally and then summarized into the following suggestions on multidimensional activities:

1. The lecture should face all emergency nurses and serve as an opportunity for professional learning.
2. The topic of writing should be identified, while the time of writing should not be required.
3. The literary level of writing is not of high significance, while the key elements of narration should be involved.
4. Interviews should be combined with writing, and the research team should have the ability to reconstruct and reply to the writing from a third perspective.
5. The period of 4 weeks may be too tight, and the period should be extended.

The above suggestions were fully considered to revise the draft MNSS. The modified activities in the first-cycle action research contained five steps, which were narrative training for nursing managers, lecture for emergency nurses (expert dimension), narrative writings and interviews (individual and expert dimensions), story rewriting and sharing (individual, colleague and expert dimensions), and group training and discussion (individual, colleague, and expert dimensions).

#### 2.4.2. Application and Improvements

The first-cycle application of the MNSS started in the first season of 2022. The narrative training and the first two lectures for emergency nurses were held in February, 2022. Owing to the outbreak of COVID-19 in Shanghai, the first cycle was postponed to July, 2022. Considering the COVID-19 prevention and control policies, the left lectures and all individual interviews were adjusted to online forms. Moreover, due to high working intensity and difficult shift scheduling in the clinical practice, participants were not forced to take part in the activities of story sharing and group training. The details of the MNSS application in the first cycle are demonstrated in the Table 1. Based on the reflection of the first cycle, several improvements on the MNSS were summarized and implemented in the second cycle.

1. Considering the difficulty in training nursing managers to analyze and rewrite stories in a professional narrative way, their narrative training can be canceled.
2. The preintervention survey should be conducted before the recruitment, and those nurses with a higher level of compassion fatigue should be enrolled.
3. Writing topics should be covered in one time, so as to make the writing coherent and compress the research time. One individual writing and one interview can be considered.
4. The topic of writing should be more focused, and a second writing with the topic of reflection can be added after story sharing.

The revised second-cycle MNSS consisted of five steps (lecture for emergency nurses, narrative writing and interview, story rewriting and sharing, reflective writing, and group training and discussion), and the details are also reported in the Table 1. Because of the different organizational structure in the second emergency department, the introduction lecture was held only once. The frequency of narrative writing and interview was reduced to improve the integrity and efficiency. Rewriting of the participants' stories required researchers' deep analysis of those writings and interviews, which were directed by narrative competence, defined as the skills required to recognize, absorb, interpret, and be moved by the stories one hears or reads [31]. When rewriting stories, the researchers combined textual, creative, and affective skills, following the procedures of identifying the original story's structure, adopting multiple perspectives, imagining interpretations and multiple endings, and entering the story's mood [31]. The process of story sharing could arouse mutual understanding between the individuals and colleagues and might function as a kind of peer support. The reflective writing provided participants an opportunity for reflection and summary. Different activities of group training organized by psychologists were attempted in two cycles to explore probably different effects.

### 2.5. Data Collection

A mixed-method data collection was performed in this study, and various data were collected prior, during, and after the MNSS using quantitative measures and qualitative methods, to facilitate the observation and reflection phases of the action research. The surveys on the effects of MNSS were conducted before (T1) and immediately after (T2) the application of MNSS with 3 instruments in the first cycle and 4 instruments in the second cycle. For better understanding of the long-term effects of the MNSS, the surveys were also implemented about 1 to 1.5 years after the MNSS (T3) concurrently among those participants in both cycles. To understand participants' evaluations on the MNSS and their perceived self-improvements, self-designed questionnaires were administered in both cycles. Meanwhile, qualitative data on participants' perceived benefits and difficulties, as well as suggestions on the MNSS, were collected through overphone interviews, group discussions, and open-ended survey questions from the participants.

The mixed-method design adopted a concurrent data analysis process, in which both quantitative and qualitative data were integrated during the analytic stage to provide a comprehensive explanation on a specific phenomenon, that was the effectiveness and feasibility of the MNSS [32]. The logical relations between the qualitative and quantitative methods in this study assumed to be complementary, where two parts of the results supplemented each other. It was supposed that those qualitative results might support and complement the quantitative findings of the MNSS's effectiveness, as well as provide sufficient feedback on the MNSS required by the action research. Consequently, the triangulation process in this study might promote complementary results from qualitative and quantitative data [32], so as to enhance the overall validity of the findings.

### 2.6. Measurements

#### 2.6.1. Professional Quality of Life

The Professional Quality of Life Scale was developed by Stamm [5]. Shen et al. [33] developed and validated the Chinese version of the scale, including a total of 25 items for three independent dimensions of compassion satisfaction, compassion fatigue, and burnout. Items were rated on a 5-point Likert response set, with two items reversely scored. A higher score indicated a higher level of the dimension. Cronbach's *α* coefficients of the dimensions ranged from 0.758 to 0.821.

#### 2.6.2. Work-Related Quality of Life

Van Laar et al. [34] developed the Work-Related Quality of Life Scale, and the 2nd edition of the scale was translated into Chinese and modified by Shao et al. [35]. The Chinese version scale consists of 7 subscales and 33 items rated on a 5-point Likert response set. The items of the Stress at Work subscale need reverse scoring to calculate the total score. A higher score indicates higher work-related quality of life. Cronbach's *α* coefficient of the Chinese scale was 0.939.

#### 2.6.3. Self-Compassion

The self-compassion scale was developed by Neff [36], and the Chinese version was validated by Chen et al. [37]. This scale consists of 26 items, contributing to six subscales. Three subscales represent positive aspects of self-compassion, while the other three assess opposite states. Items are rated on a 5-point Likert response set. The opposite items need reverse scoring to calculate the total score, and a higher score indicates higher self-compassion. Cronbach's *α* coefficient of the Chinese scale was 0.84.

#### 2.6.4. Social Support

The multidimensional scale of perceived social support was developed by Zimet et al. [38]. The Chinese version scale was translated and validated by Huang et al. [39], measuring subjectively perceived support from family, friends, and significant others (e.g., leaders and colleagues). Items are rated on a 7-point Likert response set. A higher score indicates higher perceived social support.

#### 2.6.5. Evaluations and Perceived Benefits of MNSS

The self-designed questionnaires were used to understand participants' evaluations on the MNSS and their perceived self-improvements. The number of evaluation items on the whole MNSS and rewrote stories was 12 and 6; the numbers of self-improvement items on the whole MNSS and activities of writing, story sharing, and group training were 9, 8, 12, and 7, respectively.

#### 2.6.6. Semistructured Interviews and Open-Ended Questions

Semistructured interviews were conducted with the participants, and the interview outline was designed to guide them to reflect on their experiences in the MNSS and to provide improvement suggestions. All interviews were conducted and recorded with the participants' consent by the researchers. Moreover, open-ended questions were used in the T3 survey to explore participants' perceived long-term effects, targeting on associated changes in nursing work and personal life in the past year.

### 2.7. Data Analysis

IBM SPSS 22.0 was used for statistical analysis of the collected data. Descriptive statistics were calculated to examine continuous variables ($\overset{¯}{X}$ ± *S*) and categorical factors [*n* (%)]. Repeated-measure ANOVA was used to compare the scores of involved variables across timepoints (T1, T2, and T3), so as to examine the effectiveness of the MNSS. Assumption as sphericity was tested using Mauchly's test. For those data violated sphericity, the Greenhouse–Geisser correction was adopted. Pairwise comparisons used Bonferroni corrections. The Wilcoxon signed-rank test was used to analyze the differences between the short- and long-term perceived improvements among the participants in each cycle. Statistical significance was revealed when p values were less than 0.05. Those data collected in the interviews and open-ended questions were analyzed using the Colaizzi seven-step content analysis, including the steps of familiarization, identification of meaningful statements, construction of meanings, clustering of themes, detailed description, development of basic structure, and verification of basic structure [40].

## 3. Results

### 3.1. Sample Characteristics

In both cycles, emergency nurses with different working posts and years were purposively selected to maximize the diversity and comprehensiveness of the results. In the first cycle, a total of 20 nurses agreed to participate in the MNSS. Over half (12, 60.0%) of them had the professional title of nurse, and 8 (40.0%) were senior nurses. Seven participants (35.0%) had working experiences of 1 to 3 years, seven (35.0%) with 4 to 6 years, and six (30.0%) with over 7 years. Their working posts included precheck and triage (2, 10.0%), resuscitation (2, 10.0%), infusion (9, 45.0%), intensive care (3, 15.0%), observation (3, 15.0%), and injection (1, 5.0%). Owing to the dimission of one participant, only 19 participants of the first cycle completed the long-term T3 survey. In the second cycle, 14 nurses with the score of compassion fatigue ≥2.5 agreed to participate, while one nurse dropped out owing to turnover. A total of 13 nurses participated in the MNSS and completed the surveys across three timepoints. Seven (53.8%) participants were nurses, 5 (38.5%) were senior nurses, and 1 (7.7%) was a supervisor nurse. Seven participants (53.8%) had working experiences of 1 to 3 years, four (30.8%) with 4 to 6 years, and two (15.4%) with over 7 years. Their working posts included precheck and triage (1, 7.7%), resuscitation (8, 61.5%), infusion (2, 15.4%), intensive care (1, 7.7%), and observation (1, 7.7%).

### 3.2. Descriptions of Writings and Interviews

In the first cycle, 19 participants completed two writings, with the average word counts to be (677.68 ± 308.16) and (391.95 ± 203.74). All 20 participants underwent two interviews, and the average interview time was (24.95 ± 5.09) min and (24.60 ± 7.45) min. Researchers rewrote one story for each of the 20 participants, with the average word count of (2045.65 ± 499.46). In the second cycle, 13 participants completed the writing, with the average word count of (660.77 ± 282.11), and participated in the interview, with the average interview time of (25.00 ± 5.80) min. Researchers also rewrote one story for each participant, with the average word count of (1686.46 ± 453.94). Ten participants completed the reflective writing, and the average word counts were (336.20 ± 129.89).

### 3.3. Comparison of Measurement Variables

As reported in Table 2, the scores of self-compassion among the participants increased significantly across the three timepoints in both cycles, and the T3 scores were significantly higher than those T1 scores (*P*=0.024, 0.001). In the second cycle, the scores of burnout among the participants decreased significantly across the three timepoints, while the T3 scores of burnout and compassion fatigue were both significantly lower than their T1 scores (*P* = 0.008, 0.014); the scores of work-related quality of life and perceived social support among the participants changed significantly across the three timepoints, and the T1 score of work-related quality of life was significantly lower than those T2 and T3 scores (*P* = 0.027, 0.008); besides, the T1 score of perceived social support was significantly lower than those T2 and T3 scores (*P* < 0.001, *P*=0.002), and the T2 score was significantly higher than the T3 score (*P*=0.038).

### 3.4. Evaluations and Perceived Benefits

Participants reported their evaluations on the MNSS (Table 3), as well as their perceived self-improvements (Table 4). The numbers of participants choosing specific item levels were reported. The long-term perceived improvements among the participants of the two cycles were significantly lower than their short-term perceived improvements, respectively (*Z* = −2.142, −2.499, *p*=0.032, 0.012).

### 3.5. Qualitative Data

Those data on the experiences of participants in the MNSS, as well as their perceived long-term effects of the MNSS, were reported in Tables 5 and 6.  Table 7 showed the data on suggestions that participants proposed on the MNSS.

## 4. Discussion

This study adopted the action research process and evaluated the short- and long-term effectiveness of the multidimensional narrative support system (MNSS) on emergency nurses during and after the period of COVID-19. Among those interventions targeting on the nurses' compassion satisfaction, compassion fatigue, and burnout, different outcomes were reported. A 3-minute mindfulness intervention, the 3Rs educational program and those progressive muscle relaxation exercises showed their effects on decreased compassion fatigue and burnout [14, 15, 17]. Short-term inhalation of patchouli oil might reduce emergency nurses' compassion fatigue and increase their compassion satisfaction [18]. Unlike the significant promotion on professional quality of life achieved by the compassionate mind model-based curriculum [16], a 12-week pilot of structured debriefing sessions revealed no significant differences in all three aspects before and after the interventions [20]. The different results in the two cycles of this study might be explained by the characteristics of nursing work in two units, the disparities and diversities of those shared stories, and the psychological activities of group training. Besides, during the second cycle of this action research, China optimized the COVID-19 control policies in a 10-point notice released on December, 2022. With the adjustment of policies, the rapidly increasing number of infected patients imposed additional working challenges on emergency nurses. Future research can also explore the specific effects of different activities on the participants.

Self-compassion can be regarded as “the support toward oneself when experiencing suffering or pain caused by personal mistakes and inadequacies or external life challenges” [41]. Perceived social support in this study involved those participants' subjectively assessed support from family, friends, as well as leaders and colleagues. Quantitative results proved the short- and long-term effectiveness of MNSS on its promotion of participants' self-support and perceived social support, indicating that those activities in MNSS, such as writing and interviewing, might help emergency nurses take a kinder and less judgmental approach to their sufferings [41], and story sharing and group training could provide them some extent of peer and organizational support. Results of repeated measures showed that work-related quality of life was significantly improved after the MNSS in the second cycle, indicating that the MNSS might function as an effective administrative system for the participants to achieve career growth and contribute to a favorable job environment for emergency nurses [8]. The efficiency of MNSS in the second cycle might be first attributed to the medium level of work-related quality of life before the MNSS and also confirmed its necessity when emergency nurses were exposed to unpredictable changes and stressful situations during the COVID-19 pandemic [4].

Results provided evidence that the novel support system was a scientific and logical approach refined by two cycles of action research. Besides, the MNSS was proved to be feasible and acceptable for emergency nurses, as the high portion of positive evaluations revealed that the participants showed strongly agreement on the MNSS. The outbreak of COVID-19 in Shanghai during 2022 imposed profound impact on the whole research. Consequently, the form of interviews, lectures, and sharing were adjusted to online and the time for each activity was relatively flexible considering the working schedules of most participants [22]. This strategy provided a relaxing atmosphere for participants and might not add much stress on them, which appeared to be a critical feature of the MNSS.

Participants' perceived self-improvements and qualitative data also confirmed the short- and long-term effects of MNSS. Their improved communication, reflection, and collaboration skills might facilitate their daily work in emergency departments and function as effective strategies to increase operational efficiency, improve job satisfaction and patient care, and promote comprehensive personality growth [42, 43]. The MNSS also provided a chance for participants to relieve negative emotions at work during the COVID-19 pandemic, which could reduce patient safety risks and improve health care provider well-being [44]. Comparison between participants' perceived short- and long-term self-improvements indicated that the effects of MNSS might fade over time, especially the level of social support revealed in the repeated measurement. Although long-term effects were well documented in this study, involved activities should be carried out regularly with appropriate frequency to better consolidate the effectiveness of MNSS.

After participants finished their writing and interviews, they were surprised by the experiences of healing and changing, which was consistent with the study on the storytelling through music intervention [22]. Participants proposed constructive suggestions on writing, indicating their interest in writing after the intervention. Most participants were satisfied with their rewrote stories and agreed that the rewrote story was a significant gain for them. Rewrote stories played several roles in the MNSS, including appealing for participation, summary of participants' experience, and response and support to participants. The process of organizing, exploring, and making meaning from multiple perspectives might value the context of an individual's experiences, allowing for a deeper exploration of their stories [45]. Some participants stated that they were enlightened by the rewrote stories and thought that the reorganized stories were better than their original ones.

To explore the feasibility and effects of different activities, floor curling and group talking were adopted in group training of each cycle. According to the qualitative results, the game of floor curling showed remarkable effects. Some participants believed it was a new and interesting activity for them and also an opportunity to improve the ability of teamwork. Curling is a kind of team sport that can reveal the importance of effective communication, as athletes must exactly perceive what their teammates say in the game [46]. To achieve better performance, athletes in curling should make correct and fast decisions, support each other, and cope with stress [46], which were similar with the working conditions of emergency nurses. Future MNSS should provide more various and outdoor activities for the participants.

The MNSS also features its comprehensiveness, as various activities were selected and designed from dimensions of individual, colleague, and expert. The writing process is considered as a solitary act, in which each individual must retreat into oneself, contemplate events, and imagine various meanings [24]. Based on each participant's writing, experts in humanistic sciences organized individualized interviews and reconstructed their stories, providing support for participants. During story sharing and group training, participants might feel the support from their colleagues. The peer support is a key element of the MNSS, similar with the peer-support program in which colleagues may understand present stressors more easily and have a great deal of compassion for their peers [47]. Some participants believed that peers' sharing helped them gain more perspectives and understandings and suggested sharing stories with different themes in the future.

### 4.1. Limitations and Recommendations

Several limitations should be considered in this study. As conducted during the period of COVID-19, some activities of MNSS were restricted to online forms, which might reduce the effects. Future research should explore the feasibility and effectiveness of MNSS featuring multiple face-to-face activities. This study was conducted in two tertiary general hospitals in Shanghai City, which limited its generalizability to other populations of emergency nurses. The MNSS should be promoted to other hospitals of different levels in different cities to test its applicability comprehensively. Modifications are welcomed to develop unique MNSS for different emergency departments and even other populations of clinical nurses. Moreover, competent nursing managers with narrative abilities should be cultivated.

## 5. Conclusion

This research described the development and implementation of a novel professional support system, the multidimensional narrative support system, for emergency nurses during the period of COVID-19. The five-step system integrated the narrative methods of reflective and expressive writing, as well as diverse psychological activities, and provided multiple support systems from the dimensions of colleagues and experts. The action research design was adopted in this study to modify the MNSS fully considering the participants' experiences and suggestions, which empowered the emergency nurses to refine such a support system for their own overall improvements. Despite the challenges from COVID-19, the MNSS was proved to have good feasibility, providing information about the applicability of this support system for nurses working in emergency departments. The MNSS reported a significant influence on improving emergency nurses' well-being and quality of life, and it could be recommended in more hospitals to benefit more emergency nurses.

## Figures and Tables

**Table 1:** Details of the activities in the two cycles of MNSS.

Step	First cycle			Second cycle		
	Activity	Details	Others	Activity	Details	Others
First step	Narrative training for nursing managers	Participants: nursing managers	Date: 2022.02 Duration: about 180 min	Lecture for emergency nurses	Topic: emotion management and narrative writing	Date: 2022.11 Frequency/duration: 1 time/45 min Form: online lecture
		Contents: (i) Introduction of MNSS (ii) Learning key skills of narrative nursing (iii) Arrangement of research tasks			Contents: (i) Emotions and compassion fatigue (ii) Emotion regulation and narrative writing (iii) Research introduction and recruitment	

Second step	Lectures for emergency nurses	Topic: emotion management and narrative writing	Date: 2022.02/07 Frequency/duration: 4 times/45 min for each Form: two offline and two online lectures	Narrative writing and interview	Participants: 13 participants completed writing and interview Topic of writing: an event that triggered your compassion fatigue in previous work, including your experiences and coping methods, as well as the attributions, and outcomes of the event Interview: to clarify the characters, scenes, plots, emotions, purposes, and meanings of the event; to provide support by listening, encouraging, questioning, and externalizing	Date: 2022.11–2023.01 Form: online interviews
		Contents: (i) Emotions and compassion fatigue (ii) Emotion regulation and narrative writing (iii) Research introduction and recruitment				

Third step	Narrative writings and interviews	Participants: 19 participants finished two writings; 20 completed two interviews Topic of 1st writing: an event that triggered your compassion fatigue in previous work 1st interview: to clarify the characters, scenes, plots, emotions, purposes, and meanings of the event Topic of 2nd writing: analysis of the attributions, outcomes, and coping methods of the event 2nd interview: To trigger reflection and provide support by listening, encouraging, questioning, and externalizing; to collect evaluations and suggestions	Date: 2022.07–09 Form: online interviews	Story rewriting	Participants: researchers Activity: To analyze the writings and interviews, and rewrite the event from the researchers' perspective; 13 stories were rewrote	Date: 2022.12–2023.01
				Story sharing	Participants: 11 participants, nursing researchers, assistant, manager	Date: 2023.01 Duration: about 100 min Form: online activity
					Contents: (i) Five participants voluntarily shared their own rewrote stories (ii) Other participants communicated their feelings and relevant experiences (iii) All participants completed a survey on previous activities	

Fourth step	Story rewriting	Participants: researchers Activity: to analyze the writings and interviews and rewrite the event from the researchers' perspective; 20 stories were rewrote	Date: 2022.08-09	Reflective writing	Participants: 10 participants completed the writing Topic of writing: reflection on the event and your experiences of compassion fatigue based on previous interviews, rewrote stories, and sharing experiences	Date: 2023.01-02
	Story sharing	Participants: 11 participants, nursing researchers, assistant, managers	Date: 2022.09 Duration: about 100 min Form: offline activity			
		Contents: (i) Five participants voluntarily shared their own rewrote stories (ii) Other participants communicated their feelings and relevant experiences (iii) All participants completed a survey on previous activities				

Fifth step	Group training and discussion	Participants: 9 participants, nursing researchers and assistant, and psychologists	Date: 2022.10 Duration: about 75 min Offline activity: floor curling	Group training and discussion	Participants: 7 participants, nursing researchers and assistant, and psychologists	Date: 2023.02 Duration: about 90 min Offline activity: “talking about yourself”
		Topic: team building activity/group discussion and summary			Topic: team building activity/group discussion and summary	
		Objectives: (i) To relieve work stress and negative emotions (ii) To activate the enthusiasm of teamwork (iii) To master effective communication skills (iv) To obtain support from the group and peers (v) To enhance the ability of self-regulation (vi) To discuss the experiences of and suggestions on the MNSS			Objectives: (i) To relieve work stress and negative emotions (ii) To obtain support from the group and peers (iii) To discuss the experiences of and suggestions on the MNSS	

**Table 2:** Results of repeated measures analysis in the two cycles ($\overset{¯}{X}$  ±  *S*).

Cycle	Time points	Compassion satisfaction	Burnout	Compassion fatigue	Self-compassion	Work-related quality of life	Perceived social support
First cycle (*n*_1_ = 19)	T1: pre-MNSS	3.43 ± 0.735	2.49 ± 0.584	2.44 ± 0.478	3.28 ± 0.382	3.42 ± 0.628	—
	T2: post-MNSS	3.67 ± 0.844	2.26 ± 0.639	2.24 ± 0.492	3.37 ± 0.357	3.59 ± 0.569	—
	T3: about 1–1.5 years after MNSS	3.66 ± 0.795	2.19 ± 0.535	2.24 ± 0.526	3.57 ± 0.455^a^	3.72 ± 0.620	—
	*F*	1.201	2.164	2.017	3.663	2.629	—
	*P*	0.302	0.130	0.148	0.036	0.086	—

Second cycle (*n*_2_ = 13)	T1: pre-MNSS	3.44 ± 0.590	2.96 ± 0.561	2.83 ± 0.300	3.17 ± 0.312	3.26 ± 0.721	4.70 ± 1.085
	T2: post-MNSS	3.85 ± 0.795	2.51 ± 0.588	2.74 ± 0.868	3.55 ± 0.522	3.97 ± 0.683^b^	6.22 ± 0.864^b^
	T3: about 1–1.5 years after MNSS	3.65 ± 0.620	2.26 ± 0.735^a^	2.22 ± 0.648^a^	3.69 ± 0.419^a^	3.80 ± 0.569^a^	5.66 ± 0.977^ac^
	*F*	2.098	5.861	3.449	7.786	8.532	26.818
	*P*	0.145	0.008	0.074	0.002	0.002	0.000

^a^Score at T3 was significantly different from that at T1 (*P* < 0.05). ^b^Score at T2 was significantly different from that at T1 (*P* < 0.05). ^c^Score at T3 was significantly different from that at T2 (*P* < 0.05).

**Table 3:** Evaluations on the MNSS in two cycles.

Activity	Evaluation		1^st^ cycle (*n*_1_ = 20)	2^nd^ cycle (*n*_2_ = 13)
	Items	Levels	*n*	*n*
Whole MNSS	(i) Research design was scientific and reasonable	Comparatively/completely agree	18	12
	(ii) You could understand the research purpose and theme		18	12
	(iii) Research could arouse your interests and activity		18	12
	(iv) Arrangement of research activities was logical		19	13
	(v) Arrangement of research activities showed the characteristics of narration		18	12
	(vi) Research activities could reflect the management and support from three dimensions		18	13
	(vii) Researchers showed high abilities of organization and implementation		18	12
	(viii) Research atmosphere was relaxing and pleasant, without discomfort		18	13
	(ix) Arrangement of research activities was compact, and efficiency was high		16	12
	(x) Research's overall time span (about 3 months) was relatively long		7	4
	(xi) It was of high pressure to participate in research in rest time		5	6
	(xii) You were satisfied with your performance in the research		18	10

S**t**ory rewriting	(i) How did you think the rewrote story fit your original story?	Comparatively/completely fit	19	13
	(i) Rewrote story was an important gain for your participation in this study	Comparatively/completely agree	16	11
	(ii) Reading rewrote story could deepen your understanding of compassion fatigue		16	10
	(iii) Rewrote story could reflect the researchers' understanding on you		17	12
	(i) The degree to which you approved of the rewrote story from the researcher	Comparatively/completely approve	17	13
	(i) How were you satisfied with your rewrote story?	Comparatively/completely satisfied	17	12

**Table 4:** Perceived self-improvements in the MNSS and activities in two cycles.

Self-improvements	First cycle						Second cycle				
	1^st^ writing (*n* = 19)	2^nd^ writing (*n* = 19)	Listening and sharing (*n* = 11)	Group training (*n* = 9)	Whole NMSS (short-term) (*n* = 20)	Whole NMSS (long-term) (*n* = 19)	Writing (*n* = 13)	Listening and sharing (*n* = 11)	Group training (*n* = 7)	Whole NMSS (short-term) (*n* = 13)	Whole NMSS (long-term) (*n* = 13)
	Comparatively/completely agree					Relatively/very obvious	Comparatively/completely agree				Relatively/very obvious
Improving writing skills	14	15	—	—	—	—	10	—	—	—	—
Improving expression skills	—	—	10	—	—	—	—	10	—	—	—
Improving listening skills	—	—	10	—	—	—	—	11	—	—	—
Improving levels of empathy	—	—	10	—	—	—	—	11	—	—	—
Improving professional identity and satisfaction	13	15	10	—	18	12	11	10	—	13	9
Improving emotional management skills	17	16	10	8	16	14	12	11	7	13	11
Improving skills and levels of self-compassion	16	14	10	8	18	12	10	11	7	12	9
Improving interpersonal skills	—	—	10	—	18	14	—	10	—	12	9
Improving communication skills with colleagues	—	—	—	8	—	—	—	—	7	—	—
Improving team collaboration ability	—	—	—	8	—	—	—	—	7	—	—
Improving level of perceived and received support	—	—	—	—	18	12	—	—	—	12	9
Relieving negative emotions at work	15	14	10	8	16	12	11	11	7	12	9
Reducing level of compassion fatigue	13	14	9	7	18	11	11	10	7	11	9
Reducing job burnout	14	14	10	—	16	10	11	11	—	12	6
Relieving working stress	—	—	—	8	—	—	—	—	7	—	—
Gaining resonance and support from colleagues	—	—	10	—	—	—	—	11	—	—	—
Achieving self-reflection on work	18	18	10	—	19	14	12	11	—	13	6

**Table 5:** Qualitative data on the experiences of participants.

Activity	Category	Quotations
Writing and interviewing	Relaxing opportunity to talk and express emotions	*“The interviews were relaxing and good. I could relieve my feelings in this process”*-participant 1
	Beginning to focus and reflect on own emotions	*“In the past, I would not pay attention to my emotions. When I wrote my story, I needed to notice the change of my emotions... These could help to clarify my emotions at work and in life”*-participant 4
	Helping to relieve stress at work	*“The interviews could effectively relieve the pressure at work”*-participant 22
	Feeling being healed and valued	*“When I shared my story, I could feel that I was understood, so I would like to continue and express my emotions...I could get good feedback, and the process was really healing.”* - participant 5
	Finding methods to improve future work	*“I could sort things out step by step. This method of writing can be used in my future work to help me identify and solve problems”*-participant 7

Rewrote stories and sharing	High-quality and enlightening rewrote stories	*“The rewrote stories are good and can touch on the key points... The stories can give me a sense of enlightenment”*-participant 23
	Gaining more perspectives on events	*“After sharing those negative emotions at work, I started to relax myself. I tried to understand others and avoid direct conflicts”*-participant 7
	Helping to guide future work	*“I could learn from other colleagues, like how to deal with interpersonal relationships, how to behave well in my work... It was a process of reflection”*-participant 9

Group training	Interesting activity to relieve work stress	*“The activity was very interesting, and could help me relieve the stress”*-participant 11
	Familiar with colleagues and promote team building	*“We were not familiar with each other at the beginning. But we cooperated and adjusted ourselves...We behaved like a team and made efforts to achieve the same goal... We had to organize the team quickly and effectively”*-participant 10

Whole process	Satisfied with good researchers and colleagues	*“The teachers were very responsible and the colleagues were very helpful”*-participant 22
		*“I was very satisfied with the system”*-participant 25
	Promoting oneself comprehensively	*“The process could promote our expression ability and improve ourselves... Therefore, we can treat the patients better and regulate our emotions”*-participant 12
	Reflection on previous experiences	*“It was opportunity for reflection...If we communicate together, we can find problems that we cannot see, and then we will gather together to discuss”*-participant 27

**Table 6:** Qualitative data on perceived long-term effects of participants.

Category	Quotations
Change of mentality and emotions	*“I am more optimistic. When I encounter an emergency, I can keep calm”*-participant 23
	*“Now I feel happier than when I was during the epidemic, and the atmosphere is no longer depressing”*-participant 33

Understanding of and passion for nursing work	*“I do what I can to complete my own work and help those patients. I believe “don't try to be perfect, but try your best”* -participant 30
	*“When things happen to me, I become less stubborn. I also gain new motivations for nursing work”*-participant 6

“Standing in patients' shoes”	*“When the communication with patients is not fluent, I can remind myself to stand in their shoes and be more empathetic”*-participant 19
	*“The level of my empathy, together with the interpersonal communication ability have been improved”*-participant 2

Better coping with personal emotions	*“When communicating with patients and families, I pay more attention to my tone and attitude. I can adjust negative emotions quickly, and do not bring bad emotions to other patients”*-participant 18
	*“Now I can relieve my negative emotions well and don't let those emotions influence me for too long”*-participant 23

Cultivation of thinking habits and interests	*“I will review my work and life periodically, and develop a clearer plan for my future career and life”*-participant 3
	*“I can better balance my work and life, and cultivate some interests of my own”*-participant 12

**Table 7:** Qualitative data of suggestions on the MNSS.

Activity	Category	Quotations
Writing and interviewing	Focusing on a recent event	*“If something unhappy happened recently, I could use the methods of writing and interviewing. I need to find a way to express my emotions”*-participant 8
	Providing one or different topics for writing	*“The topics of writing should be various and rich”*-participant 13
		*“We could use a case as the target to trigger the writers' memory.” Then you may find different reactions during the writing”*-participant 10
	Shortening intervals and times	*“The duration for writing and interviewing was a bit long... They should be connected more closely to help me maintain a clear mind”*-participant 11
		*“I think the two writings can be combined as one, that is, we can write a coherent writing at one time”*-participant 15
	Adding methods to relieve emotions	*“When talking about something, I may still have the emotions of anger and excitement. At this time, some methods of relief and regulation should be used”*-participant 4

Listening and sharing	Providing offline activities to promote passion	*“Online and offline activities could be combined. For example, stories can be shared online, which makes it easier for sharing. We can discuss offline to exchange feelings more directly”-*participant 28
	Sharing stories with different themes	*“Most of the shared stories had the same topic. It might be more meaningful to choose some stories with conflicting views, which could promote exploration”*-participant 22

Group training	Going outside for group activities	*“Many types of group activities can be carried out in the park...We can also try those outside activities”*-participant 16
	Providing various forms of activities	*“The activities could be more various, providing different forms of activities for the participants”*-participant 27

Whole process	Necessary to promote participants' understanding	*“It's necessary to think how to help the participants have a more open mind to participate in this activity at the beginning, and how to help them have a full understanding”*-participant 16
	Better to invite those nurses who are emotional	*“Young nurses might have more psychological changes”*-participant 12
		*“For those people who cannot relieve themselves, they can write more about these things and analyze the reasons”*-participant 16
	Providing chances to achieve immediate communication	*“If I am angry today, I can write down what happened... People who see it can respond to my writing in a timely manner”*-participant 25

## Data Availability

The data used to support the findings of this study are included within the article.
